
JOURNAL INFORMATION
==============================
NLM Title Abbreviation: Access Microbiol
Journal ID: Access Microbiol
NLM Journal ID: 101746927
Journal ID: acmi

Access Microbiology

EISSN: 2516-8290
Publisher: Microbiology Society

ARTICLE INFORMATION
==============================
PMCID: PMC8459097
PMID: 34568749
Article version: 1
Subjects: Oral Abstract

Abstracts from the Federation of Infection Societies Conference 2019

Publication date: 2020
Electronic publication date: 2020 Feb 28
Volume: 2
Issue: 2
Electronic Location ID: po0001-po0217
Copyright: © 2020 The Authors
Copyright year: 2020
License: This is an open-access article distributed under the terms of the Creative Commons Attribution License.
License URL: https://creativecommons.org/licenses/by/4.0/

==============================


Mobile Phone Contamination in Clinical Environments ‘The True Extent’

http://jmmcr.microbiologyresearch.org

Simmonds Rebecca *
Hayhust Emma
University of South Wales , Rhonda Cynon Taff , United Kingdom
*Correspondence: Rebecca simmonds, rebecca.simmonds@southwales.ac.uk

Phenotypic variation of Gardnerella vaginalis subgroups in relation to virulence potential

http://jmmcr.microbiologyresearch.org

Bulavaitė Aistė *
Janulaitienė Miglė
Baranauskienė Lina
Gėgžna Vilmantas
Plečkaitytė Milda
Institute of Biotechnology , Vilnius University , Vilnius
National Public Health Surveillance Laboratory , Vilnius , Lithuania
Institute of Biosciences , Vilnius University , Vilnius
*Correspondence: Aistė Bulavaitė, aisteb@ibt.lt

It may be a stroke, it may be meningo-encephalitis

http://jmmcr.microbiologyresearch.org

Pan Daniel *
Bridges Leslie
Cosgrove Catherine
Zhang Liqun
University of Leicester , Leicester , United Kingdom
St George's Hospital , London , United Kingdom
*Correspondence: Daniel Pan, daniel.pan@nhs.net
Print publication date: 2019 Feb
Electronic publication date: 2020 Feb 28
Volume: 2
Issue: 2
Electronic Location ID: 20
Copyright: © 2020 The Authors
Copyright year: 2020
License: This is an open-access article distributed under the terms of the Creative Commons Attribution License.
License URL: https://creativecommons.org/licenses/by/4.0/

Antimicrobial stewardship ward rounds led by junior members of the healthcare team improve patient care

http://jmmcr.microbiologyresearch.org

Hamilton Ashley Ryan *
Rogers Benedict
University Hospitals of Leicester NHS Trust , Leicester , United Kingdom
*Correspondence: Ryan Hamilton, ryan.a.hamilton@uhl-tr.nhs.uk

The link between ocular infection with non-chlamydial bacteria and trachomatous eye changes

http://jmmcr.microbiologyresearch.org

Walker-Jacobs Abigail *
Derrick Tamsyn
Ramadhani Athumani
Mafuru Elias
Massae Patrick
Malisa Aiweda
Mtuy Tara
Burton Matthew
Holland Martin
London School of Hygiene and Tropical Medicine , London , United Kingdom
King's College London , London , United Kingdom
Kilimanjaro Clinical Research Institute , Kilimanjaro Christian Medical Centre , Moshi
*Correspondence: Abigail Walker-Jacobs, abigail.walker-jacobs@kcl.ac.uk

Campylobacter lari associated Prosthetic Joint Infection

http://jmmcr.microbiologyresearch.org

Taynton Thomas *
Beard Chris
Hamilton David
York Teaching Hospitals NHS Foundation Trust , York , United Kingdom
*Correspondence: Thomas Taynton, thomas.taynton@gmail.com

Resistance trends among Enterobacterales from bacteraemias in the UK and Ireland, 2007 - 2017

http://jmmcr.microbiologyresearch.org

Aliabadi Shirin
Horner Carolyne *
Mushtaq Shazad
Livermore David
Imperial College London , London , United Kingdom
British Society for Antimicrobial Chemotherapy , Birmingham , United Kingdom
Public Health England , London , United Kingdom
University of East Anglia , Norwich , United Kingdom
*Correspondence: Carolyne Horner, chorner@bsac.org.uk

In-vitro activity of cefiderocol against multidrug-resistant Enterobacterales, Pseudomonas aeruginosa and Acinetobacter baumannii isolates from the UK

http://jmmcr.microbiologyresearch.org

Mushtaq Shazad *
Sadouki Zahra
Vickers Anna
Livermore David
Woodford Neil
Public Health England , London , United Kingdom
University College London , London , United Kingdom
University of East Anglia , Norwich , United Kingdom
*Correspondence: Shazad Mushtaq, shazad.mushtaq@phe.gov.uk

Investigating the role of the gut microbiome as a trigger for arthritis development in individuals at risk of rheumatoid arthritis

http://jmmcr.microbiologyresearch.org

Rooney Christopher *
Mankia Kulveer
Mitra Suparna
Moura Ines
Chilton Caroline
Emery Paul
Wilcox Mark
University of Leeds , Leeds , United Kingdom
Leeds Teaching Hospitals Trust , Leeds , United Kingdom
*Correspondence: Christopher Rooney, christopherrooney@nhs.net

Is hand surface coverage a good measure of hand hygiene effectiveness?

http://jmmcr.microbiologyresearch.org

Gozdzielewska Lucyna *
Price Lesley
McAloney Kareena
Lang Sue
Reilly Jacqui
Glasgow Caledonian University , Glasgow , United Kingdom
Leeds Beckett University , Leeds , United Kingdom
*Correspondence: Lucyna Gozdzielewska, lucyna.gozdzielewska@gcu.ac.uk

A diarrhoeal blast from the past

http://jmmcr.microbiologyresearch.org

Gallacher Stuart *
Khalsa Kamaljit
Deshpande Ashutosh
Ritchie Neil
Queen Elizabeth University Hospital , Glasgow , United Kingdom
*Correspondence: Stuart Gallacher, stuartgallacher@nhs.net

A decade on from the publication of the British HIV Association's National HIV Testing Guidelines: are opportunities to increase the uptake of HIV testing still being missed?

http://jmmcr.microbiologyresearch.org

Bulteel Naomi *
Bularga Anda
Baker Kerri
Sloan Derek
Banerjee Indranil
ST6 ID/GIM , NHS Fife , Kirkcaldy
Core Medical Trainee , NHS Lothian , Edinburgh
Consultant Physician in Acute & General Medicine , NHS Fife , Kirkcaldy
Consultant Infectious Diseases Physician , NHS Fife , Kirkcaldy
Senior Clinical Lecturer , University of St Andrews , St Andrews
Consultant Physician in GU Medicine & Lead Clinician for Sexual Health & HIV Service , Kirkcaldy , United Kingdom
*Correspondence: Naomi Bulteel, n.bulteel@doctors.net.uk

How can a medical educational initiative address the evolving problem of vaccine hesitancy?

http://jmmcr.microbiologyresearch.org

Costello Paul *
Bouchet Julien
Boyd Melanie
Oakley Simon
Sanofi Pasteur , Reading , United Kingdom
*Correspondence: Paul Costello, paul.costello@sanofi.com

Doxycycline for the empiric treatment of low-severity hospital acquired pneumonia

http://jmmcr.microbiologyresearch.org

Cevik Muge *
Russell Clark
Evans Morgan
Mackintosh Claire
Infection and Global Health Research , School of Medicine , University of St Andrews
NHS Lothian Infection Service , Regional Infectious Diseases Unit , Western General Hospital
University of Edinburgh Centre for Inflammation Research , Queen’s Medical Research Institute , Edinburgh
*Correspondence: Muge Cevik, mc349@st-andrews.ac.uk

Encouraging Day Three Reviews via a Pharmacist-led Antimicrobial Stewardship Ward Round

http://jmmcr.microbiologyresearch.org

Atack Kelly *
Olusoga Abimbola
Howard Philip
Leeds Teaching Hospitals NHS Trust , Leeds , United Kingdom
*Correspondence: Kelly Atack, k.atack@nhs.net

To screen or not to screen?

http://jmmcr.microbiologyresearch.org

Lowe Abigail
Sarian Arni *
Russell Katherine
Gibani Malick
Moore Luke
Mughal Nabeela
Donaldson Hugo
West Middlesex Hospital , London , United Kingdom
University of Central Lancashire , Preston , United Kingdom
Chelsea and Westminster Hospital , London , United Kingdom
*Correspondence: Arni Sarian, arni.sarian@hotmail.com

Missed opportunities for HIV testing in a Scottish Health Board

http://jmmcr.microbiologyresearch.org

Davies Melanie
Kennedy Nicholas
McGoldrick Claire *
University of Glasgow , Glasgow , United Kingdom
University Hospital Monklands , Airdrie , United Kingdom
*Correspondence: Claire McGoldrick, claire.mcgoldrick@lanarkshire.scot.nhs.uk

Investigating the microbial composition of Recurrent Vulvovaginal Candidiasis samples and biofilm formation of Candida clinical isolates

http://jmmcr.microbiologyresearch.org

McKloud Emily *
Sherry Leighann
Kean Ryan
Delaney Christopher
Metcalfe Rebecca
Ramage Gordon
University of Glasgow , Glasgow , United Kingdom
Glasgow Caledonian University , Glasgow , United Kingdom
NHS Greater Glasgow and Clyde , Glasgow , United Kingdom
*Correspondence: Emily McKloud, e.mckloud.1@research.gla.ac.uk

Rifampicin-eluting biodegradable PLGA coatings can significantly inhibit in vitro biofilm formation for 10 weeks

http://jmmcr.microbiologyresearch.org

Fraser Justine *
McCormick Christopher
Maclean Michelle
University of Strathclyde , Glasgow , United Kingdom
*Correspondence: Justine Fraser, justine.fraser@strath.ac.uk

Facial Nerve Palsy – Do people think about Lyme Disease?

http://jmmcr.microbiologyresearch.org

Fourie Benjamin
Costa Elisa
McGoldrick Claire *
University of Glasgow , Glasgow , United Kingdom
NHS Lanarkshire , Airdrie , United Kingdom
*Correspondence: Claire McGoldrick, claire.mcgoldrick@lanarkshire.scot.nhs.uk

A Paradoxical Puzzle: Delayed reaction during treatment for disseminated BCG

http://jmmcr.microbiologyresearch.org

Anpalakhan Shobana *
Schwab Uli
Newcastle upon Tyne Hospitals , Newcastle upon Tyne , United Kingdom
*Correspondence: Shobana Anpalakhan, apshobana@hotmail.com

Streptococcus agalactiae macrolide/lincosamide resistance; implications for puerperal antimicrobial therapy

http://jmmcr.microbiologyresearch.org

Davidson Alex *
Hughes Stephen
Mughal Nabeela
Moore Luke
Imperial College London , London , United Kingdom
Chelsea and Westminster NHS Foundation Trust , London , United Kingdom;
North West London Pathology , London , United Kingdom
*Correspondence: Alex Davidson, alexdavidson1995@gmail.com

Treatment Failure in a Case of Fully Susceptible Tuberculous Lymphadenitis; Time to Consider Dual Pathology

http://jmmcr.microbiologyresearch.org

Hudson Alyssa *
Jenkins Megan
Heys Izak
Moran Ed
North Bristol NHS Trust , Bristol , United Kingdom
*Correspondence: Alyssa Hudson, alyssa.clare.jacob@gmail.com

Paediatric Clostridium difficile infection: rarer than we think?

http://jmmcr.microbiologyresearch.org

Hindocha Avni *
Hatcher Jim
Lyall Hermione
Satta Giovanni
Imperial College Healthcare NHS Trust , London , United Kingdom
*Correspondence: Avni Hindocha, avni.hindocha@gmail.com

Physician-initiated HIV testing leads to missed opportunities in an East London Hospital Acute Admissions Unit

http://jmmcr.microbiologyresearch.org

Lamprianidis Konstantinos Teopoulos *
Soothill Germander Sarah
Jakubowska Agnieszka
Thomas Sherine
Barts Health NHS Trust , London , United Kingdom
*Correspondence: Konstantinos Teopoulos Lamprianidis, kteopoulos@gmail.com

Modelling Staphylococcus aureus biofilm on infected chronic wounds

http://jmmcr.microbiologyresearch.org

Cheng Yanyan *
Bank Paul De
Bolhuis Albert
University of Bath , Bath , United Kingdom
*Correspondence: Yanyan Cheng, eva928810@gmail.com

Scrub typhus causing acute undifferentiated febrile illness and its association with clinical outcomes: An observational hospital based study from tertiary care center in North India

http://jmmcr.microbiologyresearch.org

Shah Dhara *
Singh Avinash
Tiwari Dinesh
Sahu Chinmoy
Misra Richa
Prasad Kashi Nath
National Center for Disease Control , New Delhi , India
Sanjay Gandhi Post Graduate Institute of Medical Sciences , Lucknow , India
Amity Institute of Biotechnology , Lucknow , India
Apollo medics Hospital , Lucknow , India
*Correspondence: Dhara Shah, drdharashah13@gmail.com

Recurrent Urinary Tract Infections: old story, new frontiers

http://jmmcr.microbiologyresearch.org

Gewanter William *
Cliff Sheryl
Lewis Tom
Misra Soumya
North Devon District Hospital , Barnstaple , United Kingdom
*Correspondence: William Gewanter, william.gewanter@nhs.net

Post-influenza meningitis in an immunosuppressed man

http://jmmcr.microbiologyresearch.org

Senanayake Rashantha *
Burns Daniel
Canning Benjamin
Dedicoat Martin
Moran Ed
University Hospitals Birmingham , Birmingham , United Kingdom
North Bristol NHS trust , Bristol , United Kingdom
*Correspondence: Rashantha Varuni Senanayake, rvs092@gmail.com

Management of methicillin sensitive Staphylococcus aureus bacteraemia in an antimicrobial stewardship age

http://jmmcr.microbiologyresearch.org

Platts Stephen *
Payne Brendan
Price David
Schwab Ulrich
Department of Infection and Tropical Medicine , Newcastle upon Tyne Hospitals NHS Foundation Trust , Newcastle upon Tyne
School of Medical Education , Newcastle University , Newcastle upon Tyne
*Correspondence: Stephen Platts, s.platts@newcastle.ac.uk

‘The Mould that Changed the World’: Quantitative and Qualitative Analysis of Knowledge and Behavioural Change following Participation in an Antimicrobial Resistance Musical

http://jmmcr.microbiologyresearch.org

Hall Jennifer *
Jones Leah
Robertson Gail
Hiley Robin
Nathwani Dilip
Perry Meghan
Regional Infectious Diseases Unit , Western General Hospital , Edinburgh
British Society of Antimicrobial Chemotherapy , Birmingham , United Kingdom
Public Health England , Gloucester , United Kingdom
School of Mathematics , University of Edinburgh , Edinburgh
Charades Theatre Company , Edinburgh , United Kingdom
University of Dundee , Dundee , United Kingdom
*Correspondence: Jennifer Hall, j.hall15@nhs.net

Developing a quality assurance system for the use of stimulan beads in a diabetic foot clinic

http://jmmcr.microbiologyresearch.org

Burns Phillipa *
Adams Kate
Hajjawi Mark
Wearmouth Deborah
Hull University Teaching Hospitals , Hull , United Kingdom
*Correspondence: phillipa burns, phillipa.burns@hey.nhs.uk

Chronicle of migrant hyperinfective Strongyloides stercolaris larvae and associated bacteremia in a patient suffering from Idiopathic Thrombocytopenic Purpura

http://jmmcr.microbiologyresearch.org

Tak Vibhor *
Kolita Jitu Moni
Nag Vijaya Lakshmi
Bohra Gopal Krishna
Kumar Deepak
All India Institute Of Medical Sciences , Jodhpur , India
*Correspondence: Vibhor Tak, vibhor_tak@yahoo.com

Development and Initial Evaluation of a national Infection Prevention and Antimicrobial Resistance programme for UK Girlguiding and Scouts

http://jmmcr.microbiologyresearch.org

Hayes Catherine *
Eley Charlotte
Hamilton Ryan
Ashiru-Oredope Diane
McNulty Cliodna
Public Health England , Gloucester , United Kingdom
Antimicrobial Pharmacist , Leicester , United Kingdom
*Correspondence: Catherine Hayes, catherine.hayes@phe.gov.uk

Endogenous endophthalmitis as a secondary bacterial infection following viral disruption of the blood-ocular barrier?Case report of 2 renal transplant patients

http://jmmcr.microbiologyresearch.org

Price Vivien *
Li Mark
Canning Benjamin
Sandwell and West Birmingham Hospitals NHS Trust , Birmingham , United Kingdom
University Hospitals Birmingham NHS Trust , Birmingham , United Kingdom
*Correspondence: Vivien Price, vivien.price@nhs.net

Brewer’s Yeast as a cause of infective endocarditis

http://jmmcr.microbiologyresearch.org

Hammond Robert *
Aggarwal Dinesh
Hurt Will
Ferran Elena
Satta Giovanni
Abbara Aula
Imperial College Healthcare NHS Trust , London , United Kingdom
*Correspondence: Robbie Hammond, robbie.w.hammond@gmail.com

Fusobacterium nucleatum brain and liver abscesses following sigmoid diverticulum perforation in an immunocompetent patient

http://jmmcr.microbiologyresearch.org

Ghavami-Kia Bijan *
Tomlinson Ben
Li Ang
Widdrington John
Chadwick David
Mohamed Saffwan
Aziz Farooq
Martin Jessica
Kubelka Igor
James Cook University Hospital , Middlesbrough , United Kingdom
*Correspondence: Bijan Ghavami-Kia, ghavamikia@hotmail.com

Hepatitis E diagnostics: immunocompetent and IgM-negative implies PCR negative

http://jmmcr.microbiologyresearch.org

Skittrall Jordan *
Jalal Hamid
University of Cambridge , Cambridge , United Kingdom
Public Health England , Cambridge , United Kingdom
*Correspondence: Jordan Skittrall, jps55@cam.ac.uk

Is prior antibiotic exposure a risk factor for the development of bacteraemia in bone marrow transplant patients?

http://jmmcr.microbiologyresearch.org

Hills George *
Dulobdas Vaishali
Perera Nelun
Leicester Royal Infirmary , Leicester , United Kingdom
*Correspondence: George Hills, george.hills@nhs.net

Understanding antimicrobial prescribing in suspected ventilator-associated pneumonia: a prospective cohort study

http://jmmcr.microbiologyresearch.org

Docherty Callum *
White Iain
Bannard-Smith Jonathan
Morton Ben
Walters Ingeborg
Xu Yun
Roberts Stephen
McMullan Ronan
Goodacre Roy
Dark Paul
Fowler Stephen
Felton Timothy
NIHR Manchester Biomedical Research Centre , Faculty of Biology , Medicine and Health
Manchester Royal Infirmary; Manchester , United Kingdom , undefined
Clinical Sciences , Liverpool School of Tropical Medicine , Liverpool
The University of Liverpool , Liverpool , United Kingdom
Queen's University Belfast , Belfast , United Kingdom
*Correspondence: Callum Docherty, callum.docherty@student.manchester.ac.uk

Pharmacokinetics of TKM-130803 in Ebola virus disease in Sierra Leonean subjects

http://jmmcr.microbiologyresearch.org

Scott Janet *
Sharma Raman
Meredith Luke
Dunning Jake
Moore Catrin
Sahr Foday
Ward Steve
Goodfellow Ian
Horby Peter
team RAPIDE-TKM trial
MRC-University of Glasgow Centre for Virus Research , Glasgow , United Kingdom
NIHR Health Protection Research Unit in Emerging and Zoonotic Infections , Liverpool , United Kingdom
Liverpool School of Tropical Medicine , Liverpool , United Kingdom
Univ Cambridge , Dept Pathol , Cambridge
Department of Public Health , University of Makeni , Makeni
National Infection Service , Public Health England , London
Centre for Tropical Medicine and Global Health , University of Oxford , Oxford
Republic Sierra Leone Armed Forces , Military Hosp 34 , Freetown
*Correspondence: Janet Scott, janet.scott@glasgow.ac.uk

Acute haemorrhagic leukoencephalitis secondary to Chikungunya infection

http://jmmcr.microbiologyresearch.org

Jenkins Megan *
Hudson Alyssa
Heys Izak
Moran Ed
Infectious Diseases , North Bristol NHS Trust , Bristol
*Correspondence: Megan Jenkins, megan.jenkins@nbt.nhs.uk

Development of a licenced Faecal Microbiota Transplantation service for the treatment of patients in the NHS

http://jmmcr.microbiologyresearch.org

McCune Victoria *
Manzoor Susan
Shabir Sahida
Quraishi Mohammed Nabil
Sharma Naveen
Hawkey Peter
University of Birmingham Microbiome Treatment Centre , Birmingham , United Kingdom
PHE Public Health Laboratory Birmingham , Birmingham , United Kingdom
Department of Gastroenterology , University Hospitals Birmingham , Birmingham
Iqbal Tariq
*Correspondence: Mohammed Quraishi, m.n.quraishi@bham.ac.uk

Developing a PCR-Based Diagnostic Method of Detecting the Prevalence of Emerging Tick-Borne Diseases in Scotland

http://jmmcr.microbiologyresearch.org

Orr Lauren *
Glasgow Caledonian University , Glasgow , United Kingdom
*Correspondence: Lauren Orr, laurenorr@hotmail.co.uk

The Half-Life of Maternal Transplacental Antibodies in infants from mothers vaccinated with diphtheria, tetanus and pertussis: An individual participant data meta-analysis

http://jmmcr.microbiologyresearch.org

Oguti Blanche *
Assad Ali
Andrews Nick
Barug Daantje
Dang Duc Anh
Halperin Scott
Hoang Ha
Holder Beth
Kampmann Beate
Kazi Momin
Langley Joanne
Leuridan Elke
Madavan Naomi
Maertens Kirsten
Miller Elizabeth
Munoz Flor
Omer Saad
Pollard Andrew
Rice Tom
Rots Nynke
University of Oxford , Oxford , United Kingdom
Aga Khan University , Karachi , Pakistan
Public Health England , London , United Kingdom
Institute for Public Health and the Environment (RIVM) , Bilthoven , Netherlands
National Institute of Hygiene and Epidemiology , Hanoi , Vietnam
Dalhousie University , Halifax , Canada
Imperial College London , London , United Kingdom
London School of Hygiene and Tropical Medicine , London , United Kingdom
University of Antwerp , Belgium
University of Exeter , United Kingdom
Baylor College of Medicine , Houston , USA
University of Yale , New Haven , USA
*Correspondence: Blanche Oguti, blanche.oguti@paediatrics.ox.ac.uk

Case-control study of recurrent Extended-Spectrum Beta Lactamase Enterobacteriaceae Urinary Tract Infections (ESBL UTIs): the management challenges

http://jmmcr.microbiologyresearch.org

Ghani Rohma *
Mullish Benjamin
Thursz Mark
Marchesi Julian
Ghazy Anan
Davies Frances
Imperial College , London , United Kingdom
Imperial College NHS Healthcare Trust , London , United Kingdom
*Correspondence: Rohma Ghani, r.ghani@imperial.ac.uk

Cohort study of Faecal Microbiota Transplantation for patient’s colonised with MDROs - successful prevention of invasive disease despite low decolonisation rates

http://jmmcr.microbiologyresearch.org

Ghani Rohma *
Mullish Benjamin
Mcdonald Julie
Williams Horace
Gilchrist Mark
Brannigan Eimear
Satta Giovanni
Taube David
Duncan Neil
Pavlu Jiri
Ghazy Anan
Thursz Mark
Davies Frances
Marchesi Julian
Imperial College , London , United Kingdom
Imperial College NHS Healthcare Trust , London , United Kingdom
*Correspondence: Rohma Ghani, r.ghani@imperial.ac.uk

Stroke secondary to hip ulcer and Septic emboli

http://jmmcr.microbiologyresearch.org

Choudhry Taimour Rashid *
Royal Infirmary Hospital Leicester , Leicester , United Kingdom
*Correspondence: Taimour Rashid Choudhry, taimoorrashid1629@gmail.com

